# Genome-wide mRNA sequencing of a single canine cerebellar cortical degeneration case leads to the identification of a disease associated *SPTBN2* mutation

**DOI:** 10.1186/1471-2156-13-55

**Published:** 2012-07-10

**Authors:** Oliver P Forman, Luisa De Risio, Jennifer Stewart, Cathryn S Mellersh, Elsa Beltran

**Affiliations:** 1 Kennel Club Genetics Centre, Animal Health Trust, Kentford, Newmarket, Suffolk, CB8 7UU, UK; 2 Neurology Department, Animal Health Trust, Kentford, Newmarket, Suffolk, CB8 7UU, UK; 3 Centre for Preventive Medicine, Animal Health Trust, Kentford, Newmarket, Suffolk, CB8 7UU, UK

**Keywords:** Beta-III spectrin, Beagle dogs, Cerebellar cortical degeneration, Spinocerebellar ataxia type 5, Genome-wide mRNA sequencing, Cerebellum, mRNA-seq, SPTBN2, Canine, Next generation sequencing

## Abstract

**Background:**

Neonatal cerebellar cortical degeneration is a neurodegenerative disease described in several canine breeds including the Beagle. Affected Beagles are unable to ambulate normally from the onset of walking and the main pathological findings include Purkinje cell loss with swollen dendritic processes. Previous reports suggest an autosomal recessive mode of inheritance. The development of massively parallel sequencing techniques has presented the opportunity to investigate individual clinical cases using genome-wide sequencing approaches. We used genome-wide mRNA sequencing (mRNA-seq) of cerebellum tissue from a single Beagle with neonatal cerebellar cortical degeneration as a method of candidate gene sequencing, with the aim of identifying the causal mutation.

**Results:**

A four-week old Beagle dog presented with progressive signs of cerebellar ataxia and the owner elected euthanasia. Histopathology revealed findings consistent with cerebellar cortical degeneration. Genome-wide mRNA sequencing (mRNA-seq) of RNA from cerebellum tissue was used as a method of candidate gene sequencing. After analysis of the canine orthologues of human spinocerebellar ataxia associated genes, we identified a homozygous 8 bp deletion in the β-III spectrin gene, *SPTBN2*, associated with spinocerebellar type 5 in humans. Genotype analysis of the sire, dam, ten clinically unaffected siblings, and an affected sibling from a previous litter, showed the mutation to fully segregate with the disorder. Previous studies have shown that β-III spectrin is critical for Purkinje cell development, and the absence of this protein can lead to cell damage through excitotoxicity, consistent with the observed Purkinje cell loss, degeneration of dendritic processes and associated neurological dysfunction in this Beagle.

**Conclusions:**

An 8 bp deletion in the *SPTBN2* gene encoding β-III spectrin is associated with neonatal cerebellar cortical degeneration in Beagle dogs. This study shows that mRNA-seq is a feasible method of screening candidate genes for mutations associated with rare diseases when a suitable tissue resource is available.

## Background

Cerebellar cortical degeneration, also known as cerebellar abiotrophy, is a disease characterised by clinical signs of cerebellar dysfunction, such as ataxia-dysmetria, broad based stance, loss of balance and intentional tremors. Cerebellar cortical degeneration has been described in several canine breeds [1-15], classified as neonatal through to adult onset forms, with breed specific rates and extents of disease progression. In Beagles the development of neurological signs is first noticed when affected dogs start to ambulate at around three weeks of age. The affected puppies exhibit wide based-stance, loss of balance and dysmetric gait with inability to regulate rate and range of movement. Progression of the clinical signs has been reported to be minimal [2,10]. The main histopathological lesions characterising the disease in Beagle dogs are extensive degeneration to loss of Purkinje cells and secondary lesions in the molecular and granular layers [10]. A previous case report suggested an autosomal recessive mode of inheritance for neonatal cerebellar cortical degeneration (NCCD) in the Beagle [2]. Neonatal cerebellar cortical degeneration has also been reported in other canine breeds, including the Rhodesian Ridgeback, Samoyed and Irish Setter [1,9,15], with retrotransposon disruption of *GRM1* associated with neonatal cerebellar ataxia in Coton De Tulear dogs [16].

The introduction of massively parallel sequencing techniques has facilitated the use of many new DNA sequencing applications. Genome-wide sequencing such as whole genome resequencing, whole exome sequencing and genome-wide mRNA sequencing (mRNA-seq) can now be achieved in a single, cost-effective experiment. Next generation sequencing technologies coupled with target enrichment techniques [17] allows for the simultaneous sequencing of several exomes that can be used to scan coding regions of the genome for disease associated mutations in a case-control approach. An example of successful use of this method is the identification of *AFG3L2* mutations in spastic ataxia-neuropathy syndrome [18]. In the spastic ataxia-neuropathy syndrome study, DNA from just the father, mother and two affected siblings of a consanguineous family was subjected to whole exome-sequencing. Although an average of 8585 missense and 87 nonsense changes were identified per individual, use of an autosomal recessive model and exclusion of SNPs catalogued in dbSNP left just two candidate variants, only one of which was plausibly causal, illustrating the potential use of genome-wide sequencing approaches using a minimal set of individuals to identify causal variants.

Genome-wide mRNA sequencing is widely used in quantitative gene expression studies and can be used to improve genome annotation. This approach has recently been suggested for the canine genome which relies heavily on predictive methods for genome annotation [19]. Genome-wide mRNA sequencing has the potential to confirm exon boundaries, classify previously undiscovered genes, and identify gene isoforms resulting from alternative splicing, but its use as a method of identifying coding changes associated with inherited disease is not widely reported.

Following identification of a case of NCCD in a family of Beagle dogs, based on clinical presentation and histopathological findings, we undertook an mRNA-seq experiment of post-mortem cerebellar tissue. Ataxia in humans is well characterised and a large number of genes and loci have been associated with both autosomal dominant (SCA1-36) and autosomal recessive (SCAR1-12) spinocerebellar ataxias. We used the mRNA sequence data generated to interrogate canine orthologues of spinocerebellar ataxia associated genes identified from human studies with the aim of identifying the causal genetic variant responsible for NCCD in the Beagle.

## Methods

### Clinical examinations and sample collection

Three affected, full-sibling dogs from two different litters were clinically evaluated. Two dogs belonged to a first litter of seven puppies and the third affected dog belonged to a second litter of seven puppies. The affected dog from the second litter was examined at the Animal Health Trust (AHT) and the medical records and video footages of the two affected puppies from the first litter were obtained from the breeder and reviewed. The affected puppy from the second litter underwent post-mortem examination and cerebellar tissue was preserved in RNAlater (Life Technologies) for RNA extraction. Paraffin-embedded sections of cerebellum of one of the two affected puppies belonging to the first litter were available for histopathological studies. The affected puppy and its six siblings from the second litter, the dam and the sire underwent complete physical and neurological examination. All DNA samples used for *SPTBN2* mutation frequency analysis in the Beagle and other breeds had been recruited for independent investigations, and were collected from pet dogs by either buccal swabbing or from residual blood drawn as part of a veterinary procedure.

### Pathologic study

A full post-mortem was performed and the brain preserved in 10% buffered formalin for 10 days prior to processing. Upon fixation the cerebellum was transected longitudinally along the midline. The left ‘hemisphere’ was serial transected along the longitudinal axis (rostral-dorsal); the right ‘hemisphere’ was serial sectioned along the horizontal axis (medial-lateral). Cerebral serial transverse sections were taken.

Tissues were processed routinely on the Shandon Excelsior ES. Paraffin-embedded tissues were sectioned at 4–6 μm. Slides were stained on the Shandon Linistainer with Mayer’s hematoxylin and 1% alcoholic eosin. Previous slides, and their associated paraffin-embedded blocks, from the full-sibling clinically affected female puppy were retrieved for comparison and review.

### Nucleic acid preparation

Genomic DNA was extracted from whole blood samples preserved in EDTA using the Nucleon BACC2 kit (Tepnel Life Science), from buccal swabs using the QiaAmp Midi kit (Qiagen), and from formalin fixed paraffin embedded (FFPE) tissue using the Nucleospin FFPE DNA kit (Macherey Nagel). RNA was extracted using the Qiagen RNeasy Midi kit (Qiagen), including the on column DNase treatment stage. mRNA was isolated from 4.9 μg total RNA using Sera-Mag oligo(dt) particles (Thermo Fisher) and Sera-Mag mRNA isolation buffer kit (Thermo Fisher).

### mRNA-seq

Libraries were prepared using the NEBNext® mRNA Sample Prep Master Mix Set 1, consisting of RNA fragmentation, first strand cDNA synthesis, second strand synthesis, end repair, dA tailing, and PCR amplification modules. Fragmented RNA was purified using the Qiagen RNeasy mini kit (Qiagen). Reverse transcription of RNA fragments was performed using Superscript II Reverse Transcriptase (Life Technologies). Clean-up after the second strand synthesis, end repair, dA tailing, and PCR amplification modules was performed using the QIAquick PCR purification mini kit (Qiagen). The adaptor ligated library was size selected by band excision after agarose gel electrophoresis, and purified using the QIAquick gel extraction kit (Qiagen) before PCR amplification, using primers for Illumina paired-end multiplexed sequencing. The final mRNA-seq library was quantified by qPCR using the Kapa library quantification kit (Kapa Biosystems). Paired-end sequencing (51 bp reads) was carried out on a partial lane of an Illumina HiSeq2000, producing a 1.39 Gb dataset. Reads were aligned to the canine reference genome (CanFam2.0) using BWA [20]. Quality scores were re-calculated using GATK [21]. Aligned reads were viewed using The Integrative Genomics Viewer (IGV) [22].

### Polymerase chain reaction, genotyping and capillary sequencing

Polymerase chain reaction (PCR) for capillary sequencing was performed in 6 μl reactions consisting of 0.2 mM dNTPs (NEB), 1x PCR buffer (Qiagen), 0.83 μM forward primer (5’-TACTGGACACCACGGACAAGT-3’), 0.83 μM reverse primer (GGCAGAGACGTGAGTTAGCAC), 0.5 units HotStarTaq Plus DNA polymerase (Qiagen) and ultrapure water, with an expected product size of 578 bp. Cycling parameters for PCR were 95°C for 5 minutes, followed by 35 cycles of 95°C for 30 seconds, 58°C for 30 seconds and 72°C for 30 seconds, and completed with a final elongation stage of 72°C for 10 minutes. Reactions used for fragment analysis on ABI3130xl genetics analysers included 0.12 μM R110 labelled dUTP and used an alternative reverse primer (5’-GGCCTCTATCTCTGCCTTGAT-3’), for an expected product size of 268 bp. Genotyping data was analysed using Genemapper software (Applied Biosystems). 578 bp PCR products were Sanger sequenced using Big Dye v3.1 (Applied Biosystems) for capillary electrophoresis on an ABI3130xl genetic analyser. Sequencing data were analysed using Gap4 (Staden package) [23]. All primers were designed using Primer3 [24] and manufactured by IDT.

Quantitative PCR (qPCR) assays were carried out on an Illumina Eco machine in 10 μl reactions containing 5 μl Kapa Probe Fast qPCR mastermix (Kapa Biosystems), 1 x IDT PrimeTime qPCR assay mix and 2 μl cDNA (primer sequences listed in Additional file 1). Reaction efficiencies were calculated using a seven point 2 x serial dilution to create a standard curve. *SPTBN2*, *ACTB* and *TBP* reaction efficiencies were estimated at 99.3%, 94.3%, and 100.3% respectively, with standard curve r^2^ values all > 0.995.

### Immunoblotting

Canine cerebellum samples (~30 mg) were homogenised in 1 ml ice cold RIPA lysis buffer (Sigma-Aldrich), containing one complete protease inhibitor cocktail tablet per 10 ml (Roche). Protein concentrations were measured using a Qubit fluorometer (Invitrogen). Protein samples were separated by denaturing 6% SDS-PAGE (National Diagnostics). Separated proteins were transferred to a nitrocellulose membrane, which was blocked for 16 hours with 5% non-fat dried milk in phosphate-buffered saline/0.1% Tween 20 (PBS-T). Blocked nitrocellulose membranes were incubated for one hour in 1:200 goat anti-*SPTBN2* (Santa Cruz Biotechnology) or 1:1000 mouse anti-*ACTB* (Camlab) primary antibody in blocking buffer*.* After washing in PBS-T, blots were incubated in 1:10,000 HRP-conjugated donkey anti-goat or 1:1000 HRP-conjugated goat anti-mouse secondary antibody in blocking buffer. Immunoreactive proteins were detected using HRP-conjugate substrate kit for enhanced chemiluminescence.

## Results

### Clinical investigations

A four- week- old male beagle puppy presented to the AHT with ten-day history of severe cerebellar ataxia. The dog was the only affected one from a litter of seven puppies. The breeder noticed that the affected puppy was not able to ambulate normally from the onset of walking and the clinical signs had remained stable since then. The puppy was otherwise eating and drinking well and there were no signs of systemic illness in the littermates, in the dam (also during gestation) or in the sire. Physical examination did not reveal any gross abnormalities apart from the neurological signs. Neurological examination revealed severe cerebellar ataxia, with tendency to lean and fall towards both sides, resulting in inability to walk without assistance. Proprioceptive positioning was normal while hopping reactions were abnormal with delayed onset of protraction and exaggerated response, once initiated. Spinal reflexes were normal in all four limbs. Cranial nerve examination revealed an absent menace response bilaterally with normal vision. Occasionally when the head was positioned in extension spontaneous rotatory nystagmus was observed. A lesion involving mainly the cerebellum and spinocerebellar tracts was suspected. The main differential diagnoses included degenerative central nervous system disease, such as neonatal onset of cerebellar cortical degeneration and less likely inflammatory/infectious central nervous system disease, metabolic disease and neoplasia. Haematology and comprehensive biochemistry did not reveal any significant abnormalities for a four week old puppy. Brainstem auditory evoked responses identified clear waves I to V. Based on the severity of the clinical signs, normal haematology and comprehensive biochemistry, a degenerative condition was considered the most likely underlying cause and the breeder elected euthanasia. Post-mortem examination was performed an hour after euthanasia, and failed to reveal gross pathology. The brain *in toto* weighed 42 g, whilst the cerebellum weighed 5 g (12%, normal 10–12%) [2]. Narrowing of folia was not noted.

Physical and neurological examination of the dam, sire and six clinically unaffected littermates were performed at the AHT and did not reveal any abnormalities.

The only previous litter from the same dam and sire consisted of seven puppies, of which two (one female and one male) had clinical signs consistent with NCCD and the remainder were clinically normal based on clinical history and video footages provided by the breeder when the puppies were eight week old. Both clinically affected puppies were euthanized at eight weeks of age and paraffin-embedded sections of cerebellum from the female were available for histopathological examination.

### Histopathological examination results

**Figure 1:**
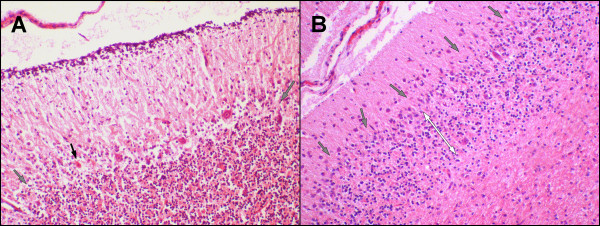
**(A) Cerebellar folia of four-week old Beagle puppy with NCCD.** Loss of occasional Purkinje cells (grey arrows). The black arrow identifies a degenerating Purkinje cell with hypereosinophilic cytoplasm and a condensed nucleus. 100x magnification. (B). Cerebellar folia of eight-week old full-sibling Beagle puppy with NCCD. Marked loss of Purkinje cells (grey arrows). Associated granular cell layer depletion secondary to Purkinje cell loss (white double headed arrows). 100x magnification.

**Figure 2:**
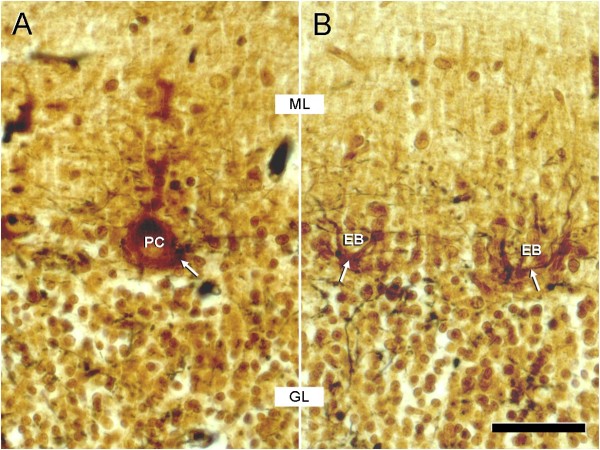
**Subacute loss of Purkinje cells (PC) is documented by so-called “empty baskets” (EB) that have been visualised by Bielschowsky´s impregnation technique.** The baskets (white arrows) synapse to the PC perikarya and in order to inhibit PC activity. ML: molecular layer; GL: granule cell layer; scale bar: 40 μm.

**Figure 3:**
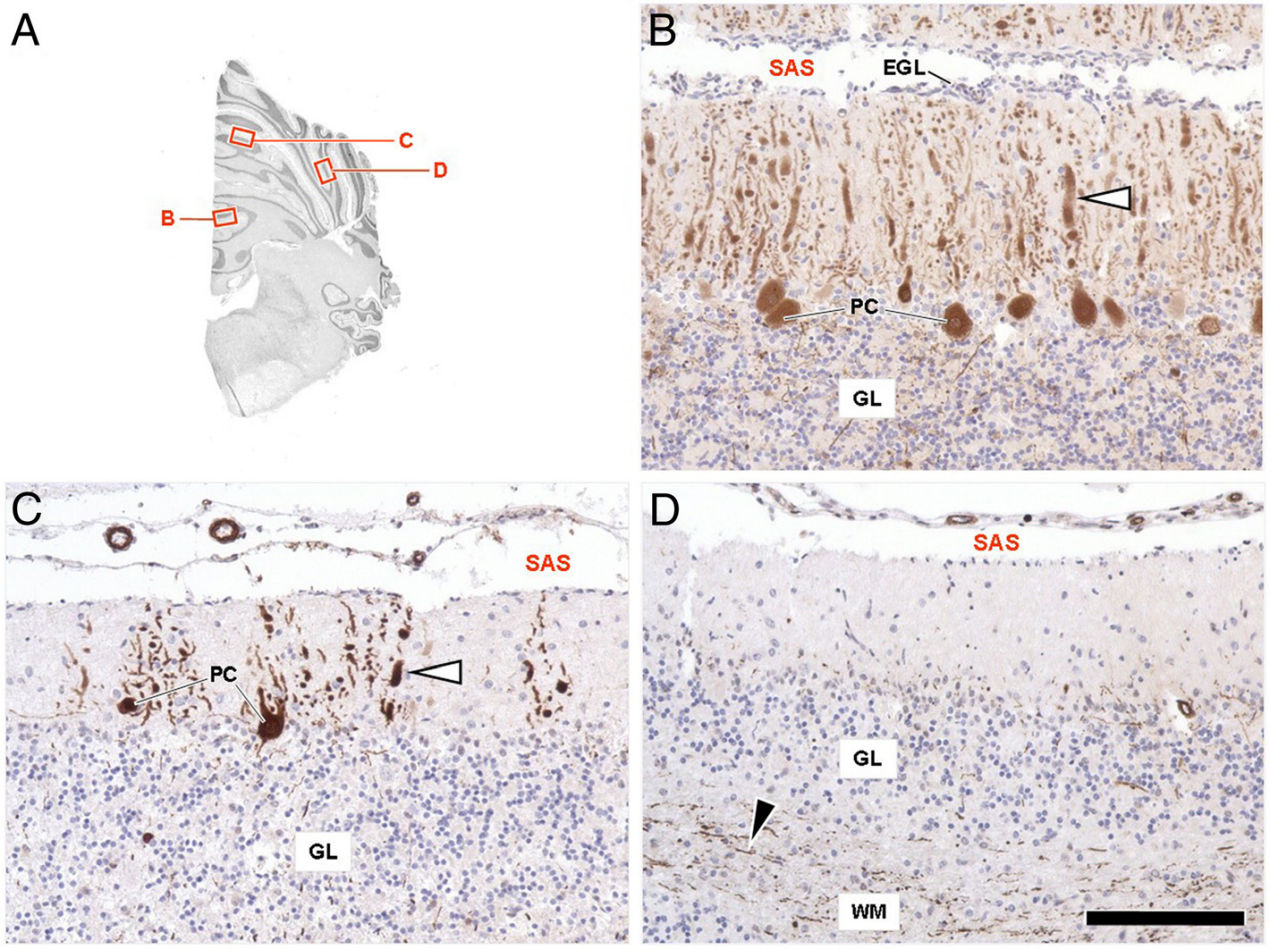
**Spatial characteristics of cerebellar cortical degeneration in the β-III spectrin deficient beagle after calbindin-immunohistochemistry and haematoxylin counterstain.(A)** Navigator figure depicting the sampled areas. **(B)** The ventral aspects of the vermis show least numeric loss of calbindin-positive (brown) Purkinje cells (PC), a narrow subarachnoid space (SAS) and remnants of the external germinative cell layer (EGL). Furthermore, the granule layer (GL) is well populated and its histoarchitecture is preserved. Degenerative changes are restricted to dystrophic dendrites (white arrowhead). Purkinje cell loss becomes increasingly evident in the dorsal vermis **(C)** and the ansiforme lobulus of the cerebellar hemispheres **(D)**. Resident PC show thickening and abnormal arborisation (C, white arrowhead) of the dendrites. Concomitantly the EGL is cytodepleted and the granular layer becomes mildly disorganised. Calbindin-staining in the most affected hemispheres is restricted to scattered axons, loosely bundled in the foliary white matter (D, black arrowhead). PC perikarya in many folia are completely missing while the Purkinje cell layer features a moderate Bergmann´s gliosis. Scale bar: 0.7 cm for A; 130 μm for B,C,D.

Histopathologically, the lesions were confined to the cerebellum. Examination of serial cerebellar sections of the four week old puppy identified mild loss of Purkinje cells, with corresponding increased numbers of astrocytes. Moderate numbers of Purkinje cells were shrunken with angular cell margins, hypereosinophilic cytoplasm, and condensed nuclei (Figure 1A). Occasional associated swollen dendritic processes were identified. Spheroids were rarely seen. Mild spongiosis was present at the granular cell layer – Purkinje cell interface Bielschowsky fiber stain was performed and demonstrated the subacute loss of Purkinje cells, also called “empty baskets” (Figure 2). Spatial characterisation after calbindin-immunohistochemistry and haematoxylin counterstain was also performed (Figure 3).

Examination of slides made from the paraffin embedded tissues of the eight week old female pup from the previous litter identified marked Purkinje cell loss with rare remaining, often abnormal, Purkinje cells (Figure 1B). Variably swollen or shrunken, hypereosinophilic Purkinje cells were identified within the remaining population. Correlating astrocytosis replaced previously lost Purkinje cells. Swollen dendritic processes and small numbers of spheroids were present. There was moderate to marked thinning of the subjacent granular layer. Cerebellar nuclei in both puppies were normal. Neuronal storage products were not identified in either puppy.

### Genetic investigations

Of a total of 14 dogs from the same sire and dam mating, 3 dogs (2 male and 1 female) were affected by NCCD, which is consistent with an autosomal recessive mode of inheritance.

**Figure 4:**
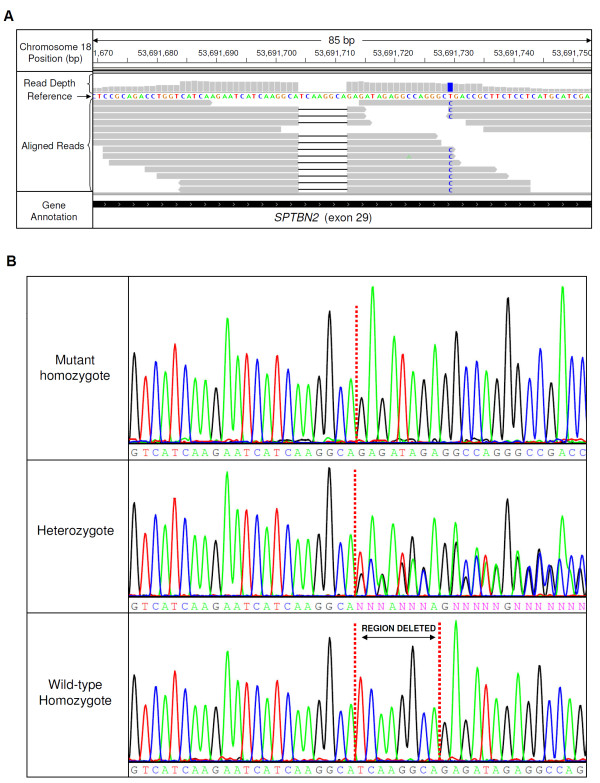
**Results of the sequencing experiments.(A)** Reads from the mRNA-seq experiment aligned across the deletion and visualised in IGV. Reads are represented by grey bars, with the deletion indicated with a black horizontal line in reads. A single nucleotide polymorphism (c.5580 T > C) is also located 18 bp downstream of the deleted sequence in the NCCD case, and is highlighted in blue. **(B)** Sanger sequencing to confirm the 8 bp deletion in the case, the sire of the case (obligate heterozygote) and a wild-type individual (sibling). The 8 bp sequence upstream of the deletion is identical to the deleted sequence.

**Figure 5:**
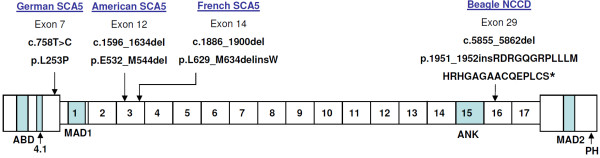
**Location of the canine 8 bp* SPTBN2 *mutation in the β-III spectrin protein.** Protein domains: ABD, actin binding domain. 4.1, protein 4.1 binding domain. ANK, ankyrin binding domain. PH, pleckstrin homology domain. MAD1; MAD2, membrane associated domains. Adapted from Bauer *et al.*[25].

A review of the scientific literature and the Online Mendelian Inheritance in Man (OMIM) database indicated 41 human ataxia loci have been identified for which 28 causal genes have been characterised. Twenty seven of the genes causing human ataxia have orthologous canine genes, and these genes were considered as candidates. The mRNA-seq experiment using cerebellum tissue from a single NCCD case produced a 1.39 Gb dataset, which has been submitted to the NBCI Sequence Reads Archive (Accession number SRA051411). The dataset consisted of 13.64 million reads, with 97.1% of the reads mapping to the dog genome. The dataset was sufficient for complete exonic coverage of 24 of the 27 candidate genes when aligned to the CanFam2 genome build. No polymorphisms were identified in 11 of the genes. Three genes contained polymorphisms in non-coding regions only. Heterozygous SNPs were identified in four genes, excluding association with NCCD. Two genes contained synonymous SNPs. Non-synonymous changes were identified in *ITPR1* (chr20:15,780,361 G > C;p.E2491Q), *BEAN1* (chr5:85,782,181 C > T;p.R247Q), and *ADCK3* (chr7:41,059,467A > G;p.S328P). For *ITPR1* and *ADCK3* the non-reference residue is highly conserved amongst vertebrate species and therefore could be ruled out as causal. Alignment across vertebrate species at the site of the *BEAN1* polymorphism indicates that both glutamic acid and glutamine residues occur naturally, enabling that variant to also be excluded. An 8 bp deletion was detected in exon 29 of *SPTBN2,* chr18:53,691,704_53,691,711del, and is shown in Figure 4A. The deletion was confirmed by Sanger sequencing (Figure 4B). The frameshift is predicted to result in a run of 27 aberrant amino acids, followed by premature termination with a 410 amino acid truncation p.G1952insRDRGQGRPLLLMHRHGAGAACQEPLCS*. The mutation is located in the 16^th^ of 17 spectrin repeat domains located in β-III spectrin (Figure 5). A summary of candidate genes and variants is shown in Additional file 2.

**Figure 6:**
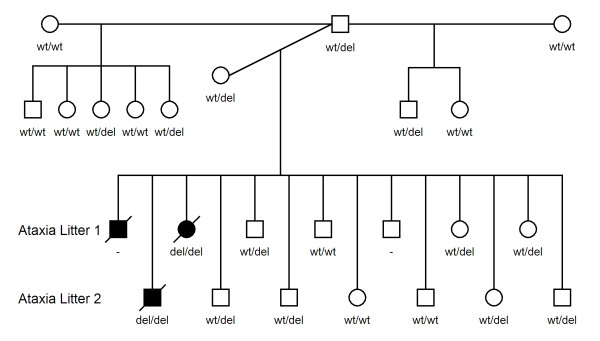
**Pedigree of the NCCD Beagle family.** Squares and circles represent male and female individuals respectively. Shaded symbols represent NCCD cases (all deceased). The distribution and number of affected individuals is consistent with an autosomal recessive mode of inheritance. Homozygous wild-type individuals are represented as wt/wt, heterozygotes as wt/del, and mutant homozygotes as del/del.

Genotyping experiments were performed to establish whether the 8 bp *SPTBN2* deletion could be potentially causal. The sire and dam of the affected dogs were both heterozygous for the 8 bp deletion, and out of the ten clinically unaffected siblings tested, seven were heterozygous and three were homozygous for the wild-type allele. DNA extracted from FFPE tissue of a previous NCCD case from the same sire and dam mating genotyped homozygous for the deletion. Seven other clinically unaffected half-siblings, with the same sire as the affected dogs, were either heterozygous or wild-type homozygous. An extended pedigree is shown in Figure 6. An additional 145 Beagles, which were collected for an unrelated project and clinically normal with respect to NCCD, were also genotyped. Eight dogs were heterozygous for the deletion, and the remaining 137 dogs were homozygous wild-type, in full concordance with the mutation being causal. In addition 513 dogs from 37 other breeds were also genotyped; all were homozygous for the wild-type allele.

**Figure 7:**
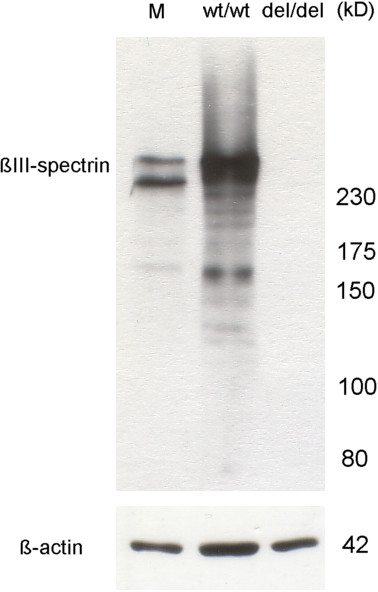
**Western blot analysis of wild-type and NCCD affected cerebellum tissue homogenates.** Full length β-III spectrin was confirmed in wild-type cerebellum tissue (wt/wt), but no full length or truncated β-III spectrin could be identified in cerebellum tissue from the NCCD affected Beagle (del/del). Mouse brain extract (M) was used a positive control. Beta-actin was used as a loading control.

Limited qPCR experiments to assess the expression levels of *SPTBN2* in affected and normal cerebellum tissues suggested a 68 fold reduction in *SPTBN2* transcript levels in the affected Beagle (data shown in Additional file 3). Using Western blot analysis with primary antibodies targeting the N-terminal region of β-III spectrin, no full length or truncated β-III spectrin could be detected in cerebellum tissue of the NCCD case, suggesting expression of the protein may have been abolished (Figure 7).

## Discussion

In this article we have described NCCD in three Beagle dogs and report the use of genome-wide mRNA sequencing of a single case to identify an 8 bp deletion in the β-III spectrin gene, *SPTBN2*, that segregates consistently with the disorder. We have shown that the use of mRNA-seq for candidate gene analysis can potentially remove the need for a genome-wide association study (GWAS) stage, an approach commonly and successfully used in the dog even with small case-control sets, reducing cost and shortening study time-frames. Because the mRNA-seq approach requires far fewer samples than the GWAS approach, less time is needed in the sample collection phase, allowing projects to commence earlier, especially when cases are rare. To our knowledge this is the first report of this application for mRNA-seq. The approach has advantages over exome enrichment and sequencing methodology, in that the method can be performed in all species with reference genome sequence builds, without the need for a proprietary kit. The mRNA-seq approach is also not dependant on reference genome annotation, which may be inaccurate or incomplete in some species.

Initial clinical presentation and progression of the three affected Beagle puppies were highly suggestive of NCCD, with histopathological studies confirming Purkinje cell degeneration and necrosis in different stages according to disease progression in the two puppies that underwent necropsy. Histologically both the four-week old puppy and eight-week old puppy revealed Purkinje cell degeneration and loss. Lesions present in the eight-week old puppy demonstrated disease progression expected with the prolonged survival time, with increased proportions of lost Purkinje cells, and the presence of corresponding degeneration of the granular cell layer. Changes in both puppies were limited to Purkinje cell death and secondary changes associated with the loss of Purkinje cells (ie astrocytosis, granular cell degeneration, axonal swelling). Regional variation, as previously described by Yasuba et al., was not identified [10], but all the affected Beagles in that study were euthanized at 14 weeks of age, and the regional variation could just be related to the progression of the disease.

The identification of an 8 bp deletion in *SPTBN2*, a gene associated with spinocerebellar type 5 (SCA5) in humans [26], that fully segregates with the disease provides a strong candidate variant for NCCD in the Beagle. Spectrins are a family of cytoskeletal proteins, with tetrameric structures comprising two α and two β subunits, with diversity and specialisation of function. Spectrins are important structural components of the plasma membrane and play a significant role in restricting and stabilising membrane spanning proteins within specific subdomains of the plasma membrane. The spectrin cytoskeleton was first discovered in erythrocytes and has since been identified in a variety of cells [27]. β-III spectrin is primarily expressed in the nervous system and the highest levels of expression are found in Purkinje cell soma and dendrites [28]. β-III spectrin has been shown to stabilise the glutamate transporter *EAAT4* at the plasma membrane of the Purkinje cells [29], facilitate protein trafficking by linking the microtubule motor to vesicle-bound cargo [30] and maintain a high density of sodium channels within the soma and dendrites of Purkinje cells [31]. β-III spectrin is critical for development of Purkinje cells [32].

In humans, three mutations in *SPTBN2* have been shown to cause autosomal dominant SCA5. The identified causal mutations include two in-frame deletions of 39 and 15 bp which alter the structure of the 3^rd^ of 17 spectrin repeats, and a single base pair substitution causing an amino acid change (L253P) in a highly conserved region of the calponin homology domain [26]. The consequence of the two in-frame deletions in β-III spectrin is predicted to be disruption of the highly ordered triple alpha helical structure of the spectrin repeat, causing conformational changes in the tetrameric α-β spectrin complex [26]. Studies suggest the resulting mutant protein may affect the localisation of EAAT4 and GluRδ2, one possible outcome of which is glutamate signalling abnormalities and Purkinje cell death [26]. The L253P missense mutation has been shown to result in loss of interaction with the Arp1 subunit of the dynactin-dynein complex, affecting the role of β-III spectrin in vesicle trafficking, preventing transport of both β-III spectrin and EAAT4 to the cell membrane from the Golgi apparatus in Purkinje cells causing cell dysfunction and death. [33]

Experimentally induced β-III spectrin deficiency in mice from two independent studies resulted in phenotypes that resemble NCCD in Beagle dogs [31,34]. One β-III spectrin deficient strain was produced by targeting replacement of exon 3 to 6 of *SPTBN2* with the neomycin-resistance gene*,* resulting in a frameshift and a premature stop codon in exon 7. As a result no full length β-III spectrin is produced in -β-III^−/−^ mice, although a low level of near full length protein is produced due to novel exon 1 (rather than exon 2) to exon 7 splicing [31]. Homozygous β-III spectrin deficient mice develop characteristics of progressive cerebellar ataxia from a few weeks of age with cerebellar atrophy and Purkinje cell loss. In the parallel study the β-III spectrin deficient mouse strain is the result of βgeo insertion between exons 25 and 26 resulting in premature termination in spectrin repeat 14, which is closer to the position to the Beagle mutation, although results in the loss of the ankyrin binding domain [34]. The β-III spectrin deficient mice from this study display a mild non-progressive ataxia by 6 months and a myoclonic seizure disorder by one year [34]. It is apparent that onset of ataxia is later for the β-III spectrin deficient mouse in comparison to Beagle NCCD cases, with mice not showing significant signs of ataxia until six months of age (past sexual maturity). This is more comparable to the human disease, though the differences in the modes of inheritance suggest different mutational effects. Deficient mice also show only a mild ataxia and remain ambulatory, while the dogs described are more severe both in terms of degree of ataxia (astasia) and Purkinje cell loss. In the study by Stankewich *et al.*, no Purkinje cell loss was documented by 18 months of age, only atrophy of the dendritic arbor. Disparity in phenotype between species may suggest differences in cerebellar development, function, and potentially the involvement of β-III spectrin. Further understanding of canine cerebellar function would be required to shed light on the described differences and common principles.

It has been shown that heterozygous mice, generated by exon 2–6 replacement, do not display any characteristics of cerebellar ataxia [33], in common with heterozygous dogs in the Beagle population, suggesting that SCA5 in heterozygous humans is caused by dominant negative effects of mutant β-III spectrin, rather than haploinsufficiency. Histopathologic examination in heterozygous mice revealed normal size and morphology of the cerebellum and immunostaining studies showed no changes on Purkinje cell morphology. These histopathological findings cannot be correlated with heterozygous Beagle dogs as none underwent post-mortem examination and all of them are currently alive and clinically unaffected. Interestingly, slight motor impairments were reported for heterozygous mice generated by βgeo insertion between exons 25 and 26, perhaps indicating that the truncated protein is having a slight dominant negative effect, and illustrates how disease progression is dependent on the positioning of *SPTBN2* mutations.

The 8 bp deletion in the dog is located at a tandem repeat sequence, suggesting homologous recombination as the deletion mechanism. The position of a SNP (c.5580 T > C) 18 bp downstream of the deleted sequence removes a possible termination site for the mutant protein and extends the sequence of potential aberrant amino acids from 6 to 27. Although expression analysis was limited, due to the availability of only one case and one control, results are suggestive of a 68 x reduction in the relative levels of *SPTBN2* in the NCCD case cerebellum, which may be due to nonsense mediated decay. Even though *SPTBN2* expression is greatly reduced in NCCD affected cerebellum tissue, sufficient read depth from the mRNA-seq expression was still achieved, because of the high levels of *SPTBN2* expression normally seen in cerebellum tissue. Further to a reduction in mRNA levels, no full length or truncated β-III spectrin was detectable in NCCD affected cerebellum tissue by Western blot analysis. This may indicate that the 8 bp deletion results in a full knock-out of *SPTBN2*. A full gene knock-out eliminates the possibility of a dominant negative effect that could be caused by a truncated form of the β-III spectrin protein, which is in agreement with heterozygous dogs showing no clinical signs.

Although NCCD is likely to be heterogeneous in different canine breeds, screening for the *SPTBN2* deletion in non-beagle cases has not been investigated to confirm this. It is possible that the mutation could exist at very low frequencies in other breed populations, especially those closely related to the beagle, but extensive screening of large numbers of individuals would be required to fully investigate this possibility.

## Conclusion

This study demonstrated that a spontaneous canine model of spinocerebellar ataxia type 5 is caused by a mutation in *SPTBN2*, the gene encoding β-III spectrin, which offers an invaluable opportunity for further understanding of the disease pathogenesis and treatment. The Beagle is a naturally occurring, animal model for SCA5 and may help to increase understanding of disease progression in humans. This study also shows that mRNA-seq sequencing is a feasible method to identify mutations associated with rare diseases when a suitable tissue resource is available. A genetic test has been developed to identify carriers and to eradicate the disease from the breed.

## Competing interests

Patent pending on the use of the *SPTBN2* locus as a canine diagnostic marker.

## Authors’ contributions

EB diagnosed the NCCD case, performed neurological work-ups and coordinated sample collection from the case and relatives. OPF conceived mRNA-seq experiments, prepared sequencing libraries and analysed all sequencing data. OPF performed expression analysis and protein work. JS performed the histopathological examinations. OPF, EB and JS all contributed sections to the manuscript. CSM and LDR advised on the study and revised the manuscript. All authors approved the final manuscript.

## Supplementary Material

Additional file 1 **Primers used for qPCR assays of the*SPTBN2*,*ACTB and TBP*genes.** All probes were 5’ 6-FAM and 3’ Iowa Black labelled, with internal ZEN labelling. Click here for file

Additional file 2 Summary of candidate genes investigated for sequence polymorphisms after mRNA-seq of a single NCCD Beagle case. Click here for file

Additional file 3 **Relative expression analysis data.** Expression levels of *SPTBN2* were measured relative to *ACTB* and *TBP* using qPCR. Fold change was calculated based on changes in threshold cycle (Ct) measurements within (ΔCt) and between (ΔΔCt) the case and control. Click here for file

## References

[B1] de Lahunta A Glass E Veterinary Neuroanatomy and Clinical Neurology 2008 3 Saunders, Philadelphia 348 388

[B2] Kent M Glass E de Lahunta A Cerebellar cortical abiotrophy in a beagle J Small Anim Pract 2000 41 7 321 323 10.1111/j.1748-5827.2000.tb03210.x 10976629

[B3] Jokinen TS Cerebellar cortical abiotrophy in Lagotto Romagnolo dogs J Small Anim Pract 2007 48 8 470 473 10.1111/j.1748-5827.2006.00298.x 17490444

[B4] Olby N Cerebellar cortical degeneration in adult American Staffordshire Terriers J Vet Intern Med 2004 18 2 201 208 10.1111/j.1939-1676.2004.tb00161.x 15058771

[B5] Gandini G Cerebellar cortical degeneration in three English bulldogs: clinical and neuropathological findings J Small Anim Pract 2005 46 6 291 294 10.1111/j.1748-5827.2005.tb00323.x 15971900

[B6] Flegel T Cerebellar cortical degeneration with selective granule cell loss in Bavarian mountain dogs J Small Anim Pract 2007 48 8 462 465 10.1111/j.1748-5827.2006.00257.x 17663663

[B7] Speciale J de Lahunta A Cerebellar degeneration in a mature Staffordshire terrier J Am Anim Hosp Assoc 2003 39 5 459 462 14518653 10.5326/0390459

[B8] Steinberg HS Cerebellar degeneration in Old English Sheepdogs J Am Vet Med Assoc 2000 217 8 1162 1165 10.2460/javma.2000.217.1162 11043686

[B9] Chieffo C Cerebellar Purkinje's cell degeneration and coat color dilution in a family of Rhodesian Ridgeback dogs J Vet Intern Med 1994 8 2 112 116 10.1111/j.1939-1676.1994.tb03207.x 8046673

[B10] Yasuba M Cerebellar cortical degeneration in beagle dogs Vet Pathol 1988 25 4 315 317 10.1177/030098588802500412 3407102

[B11] Varejao AS Prada J Rodrigues PA Cerebellar cortical degeneration in two Azores Cattle Dog littermates J Small Anim Pract 2008 49 7 371 18638062 10.1111/j.1748-5827.2008.00616.x

[B12] Urkasemsin G Hereditary cerebellar degeneration in Scottish terriers J Vet Intern Med 2010 24 3 565 570 10.1111/j.1939-1676.2010.0499.x 20384950

[B13] Thomas JB Robertson D Hereditary cerebellar abiotrophy in Australian kelpie dogs Aust Vet J 1989 66 9 301 302 10.1111/j.1751-0813.1989.tb13959.x 2818374

[B14] Gumber S Cho DY Morgan TW Late onset of cerebellar abiotrophy in a boxer dog Vet Med Int 2010 2010 406275 21151662 10.4061/2010/406275 PMC2997505

[B15] Coates JR Neonatal cerebellar ataxia in Coton de Tulear dogs J Vet Intern Med 2002 16 6 680 689 10.1111/j.1939-1676.2002.tb02408.x 12465765

[B16] Zeng R A truncated retrotransposon disrupts the GRM1 coding sequence in Coton de Tulear dogs with Bandera's neonatal ataxia J Vet Intern Med 2011 25 2 267 272 10.1111/j.1939-1676.2010.0666.x 21281350

[B17] Asan NF Comprehensive comparison of three commercial human whole-exome capture platforms Genome Biol 2011 12 9 R95 10.1186/gb-2011-12-9-r95 21955857 PMC3308058

[B18] Pierson TM Whole-Exome Sequencing Identifies Homozygous AFG3L2 Mutations in a Spastic Ataxia-Neuropathy Syndrome Linked to Mitochondrial m-AAA Proteases PLoS Genet 2011 7 10 e1002325 10.1371/journal.pgen.1002325 22022284 PMC3192828

[B19] Derrien T Annotation of the domestic dog genome sequence: finding the missing genes Mamm Genome 2011 23 Numbers 1-2 (2012) 124 131 22076420 10.1007/s00335-011-9372-0

[B20] Li H Durbin R Fast and accurate short read alignment with Burrows-Wheeler transform Bioinformatics 2009 25 14 1754 1760 10.1093/bioinformatics/btp324 19451168 PMC2705234

[B21] McKenna A The Genome Analysis Toolkit: a MapReduce framework for analyzing next-generation DNA sequencing data Genome Res 2010 20 9 1297 1303 10.1101/gr.107524.110 20644199 PMC2928508

[B22] Robinson JT Integrative genomics viewer Nat Biotechnol 2011 29 1 24 26 10.1038/nbt.1754 21221095 PMC3346182

[B23] Bonfield JK Smith K Staden R A new DNA sequence assembly program Nucleic Acids Res 1995 23 24 4992 4999 10.1093/nar/23.24.4992 8559656 PMC307504

[B24] Rozen S Skaletsky H Primer3 on the WWW for general users and for biologist programmers Methods Mol Biol 2000 132 365 386 10547847 10.1385/1-59259-192-2:365

[B25] Bauer P Schols L Riess O Spectrin mutations in spinocerebellar ataxia (SCA) Bioessays 2006 28 8 785 787 10.1002/bies.20443 16927298

[B26] Ikeda Y Spectrin mutations cause spinocerebellar ataxia type 5 Nat Genet 2006 38 2 184 190 10.1038/ng1728 16429157

[B27] Bennett V Baines AJ Spectrin and ankyrin-based pathways: metazoan inventions for integrating cells into tissues Physiol Rev 2001 81 3 1353 1392 11427698 10.1152/physrev.2001.81.3.1353

[B28] Sakaguchi G A novel brain-specific isoform of beta spectrin: isolation and its interaction with Munc13 Biochem Biophys Res Commun 1998 248 3 846 851 10.1006/bbrc.1998.9067 9704016

[B29] Jackson M Modulation of the neuronal glutamate transporter EAAT4 by two interacting proteins Nature 2001 410 6824 89 93 10.1038/35065091 11242047

[B30] Holleran EA beta III spectrin binds to the Arp1 subunit of dynactin J Biol Chem 2001 276 39 36598 36605 10.1074/jbc.M104838200 11461920

[B31] Perkins EM Loss of beta-III spectrin leads to Purkinje cell dysfunction recapitulating the behavior and neuropathology of spinocerebellar ataxia type 5 in humans J Neurosci 2010 30 14 4857 4867 10.1523/JNEUROSCI.6065-09.2010 20371805 PMC2857506

[B32] Gao Y beta-III spectrin is critical for development of purkinje cell dendritic tree and spine morphogenesis J Neurosci 2011 31 46 16581 16590 10.1523/JNEUROSCI.3332-11.2011 22090485 PMC3374928

[B33] Clarkson YL Beta-III spectrin mutation L253P associated with spinocerebellar ataxia type 5 interferes with binding to Arp1 and protein trafficking from the Golgi Hum Mol Genet 2010 19 18 3634 3641 10.1093/hmg/ddq279 20603325 PMC2928133

[B34] Stankewich MC Targeted deletion of betaIII spectrin impairs synaptogenesis and generates ataxic and seizure phenotypes Proc Natl Acad Sci U S A 2010 107 13 6022 6027 10.1073/pnas.1001522107 20231455 PMC2851889

