# Supplementary material for: Genome-wide mRNA sequencing of a single canine cerebellar cortical degeneration case leads to the identification of a disease associated SPTBN2 mutation
Source: BMC Genet. 2012 Jul 10;13:55. doi: 10.1186/1471-2156-13-55 (PMC3413603; doi:10.1186/1471-2156-13-55)
Supplement: Additional file 1 — Primers used for qPCR assays of theSPTBN2,ACTB and TBPgenes. All probes were 5’ 6-FAM and 3’ Iowa Black labelled, with internal ZEN labelling. [file 1471-2156-13-55-S1.doc]

**Additional file 1**

Primers used for qPCR assays of the *SPTBN2*, *ACTB and TBP* genes. All probes were 5’ 6-FAM and 3’ Iowa Black labelled, with internal ZEN labelling.

| **Assay name** | **Forward primer sequence** | **Probe sequence** | **Reverse primer sequence** | **size (bp)** |
| --- | --- | --- | --- | --- |
| SPTBN2 | TGGATGGTGAAGAGCAGAAC | TTCTCAGTGAACTTGGGTGGCTTCTC | TCCTTCAATTCCTATCGCACG | 83 |
| ACTB | CCAACCGTGAGAAGATGACC | CGAGACTTTCAACACCCCAGCCA | CGTACAGGGACAGCACAG | 90 |
| TBP | TCTGGCATATTTCCTCGCTG | ACTGTTCTTCACTCTTGGCTCCCG | TTCAGTTCTGGGAAGATGGTG | 78 |
