# Supplementary material for: Genome-wide mRNA sequencing of a single canine cerebellar cortical degeneration case leads to the identification of a disease associated SPTBN2 mutation
Source: BMC Genet. 2012 Jul 10;13:55. doi: 10.1186/1471-2156-13-55 (PMC3413603; doi:10.1186/1471-2156-13-55)
Supplement: Additional file 2 — Summary of candidate genes investigated for sequence polymorphisms after mRNA-seq of a single NCCD Beagle case. [file 1471-2156-13-55-S2.doc]

**Additional file 2**

Summary of candidate genes investigated for sequence polymorphisms after mRNA-seq of a single Beagle NCCD case.

| Ataxia type | Associated gene | Human Loci | GRCh37/hg19 region | CanFam2 syntenic region | Notes |
| --- | --- | --- | --- | --- | --- |
| SCA1 | *ATXN1* | 6p22.3 | chr6:16,299,344-16,761,721 | chr35:18,460,465-18,865,962 | Heterozygous SNPs identified - gene excluded |
| SCA2 | *ATXN2* | 12q24.12 | chr12:111,890,019-112,037,480 | chr26:12,091,459-12,206,061 | No polymorphisms identified |
| SCA3 | *ATXN3* | 14q32.12 | chr14:92,524,897-92,572,965 | chr8:4,316,475-4,352,391 | No polymorphisms identified |
| SCA4 | *-* | 16q22.1 | chr16:66,700,000-70,800,000 | chr5:79,600,000-85,600,000 | n/a |
| SCA5 | *SPTBN2* | 11q13.2 | chr11:66,452,720-66,488,870 | chr18:53,664,195-53,696,100 | 8 bp deletion exon 29. Frameshift. Prediction: 27 Aberrant amino acids, 410 amino acid truncation (p.I1953Rfs*28). |
| SCA6 | *CACNA1A* | 19p13.2 | chr19:13,317,256-13,617,274 | chr20:51,822,254-52,037,590 | Heterozygous SNPs identified - gene excluded |
| SCA7 | *ATXN7* | 3p14.1 | chr3:63,850,233-63,982,293 | chr20:30,222,502-30,361,703 | No polymorphisms identified |
| SCA8 | *ATXN8OS* | 13q21.33 | chr13:70,681,345-70,713,885 | chr22:27,500,593-27,536,451 | Insufficient read depth to analyse |
| SCA9 | *-* | *-* | *-* | *-* | n/a |
| SCA10 | *ATXN10* | 22q13.31 | chr22:46,067,678-46,241,187 | chr10:23,331,497-23,495,457 | No polymorphisms identified |
| SCA11 | *TTBK2* | 15q15.2 | chr15:43,036,542-43,213,007 | chr30:12,667,929-12,836,871 | Heterozygous SNPs identified - gene excluded |
| SCA12 | *PPP2R2B* | 5q32 | chr5:145,969,068-146,461,033 | chr2:43,854,239-44,295,609 | No polymorphisms identified |
| SCA13 | *KCNC3* | 19q13.33 | chr19:50,818,765-50,832,634 | - | No canine orthologue |
| SCA14 | *PRKCG* | 19q13.42 | chr19:54,385,467-54,410,901 | chr1:106,377,729-106,388,206 | No polymorphisms identified |
| SCA15 | *ITPR1* | 3p26.1 | chr3:4,535,032-4,889,524 | chr20:15,748,595-16,067,260 | 4 Exonic SNPs. 1 Non-synonymous SNP (p.E2491Q). Q residue conserved across mammalian species |
| SCA16 (see SCA15) | *-* | - | - | - | n/a |
| SCA17 | *TBP* | 6q27 | chr6:170,863,471-170,881,946 | chr12:75,485,739-75,496,000 | No polymorphisms identified |
| SCA18 | *IFRD1** | 7q22-q32 | chr7:112,063,199-112,117,258 | chr14:59,850,326-59,898,828 | No coding polymorphism identified |
| SCA19 | *-* | *1p21-q21* | *chr1:94,700,000 - 155,000,000* | *multiple chromosomes* | n/a |
| SCA20 | 12 gene duplication. | *11q12* | *chr11:61,453,940-61,746,519* | *chr18:57,759,467-57,508,030* | n/a |
| SCA21 | - | 7p21.3-p15.1 | ch7:10,075,254-28,198,059 | multiple chromosomes | n/a |
| SCA22 | *-* | *1p21-q21* | *chr1:94,700,000 - 155,000,000* | *multiple chromosomes* | n/a |
| SCA23 | *PDYN* | *20p13* | *chr20:1,959,402-1,974,931* | *chr24:22,050,982-22,054,521* | Insufficient read depth to analyse |
| SCA24 (see SCAR4) | *-* | *-* | *-* | *-* | n/a |
| SCA25 | *-* | *2p21-p13* | *chr2:41,800,000-75,000,000* | *chr10,chr17* | n/a |
| SCA26 | *-* | *19p13.3* | *chr19:998,644-4,392,667* | *chr20:58,226,442-60,850,479* | n/a |
| SCA27 | *FGF14* | *13q33.1* | *chr13:102,373,205-103,054,124* | *chr22:54,328,225-54,929,598* | No coding polymorphisms identified. |
| SCA28 | AFG3L2 | 18p11.21 | chr18:12,328,943-12,377,275 | chr7:80,792,562-80,830,840 | No polymorphisms identified |
| SCA29 | *-* | *heterogeneous* | *-* | *-* | n/a |
| SCA30 | *-* | *4q34.3-q35.1* | *chr4:179,213,356-184,216,082* | *chr16:47,000,000-53,000,000* | n/a |
| SCA31 | *BEAN1* | *16q21* | *chr16:66,460,816-66,527,432* | *chr5:85,780,302-85,806,244* | 1 non synonymous exonic SNP (p.R247Q). Conservation data suggests R or Q acceptable. |
| SCA32 | *-* | *7q32-q33* | *chr7:131125523-132115310* | *chr14 77,000,000-86,000,000* | n/a |
| SCA33 | *not characterised* | *-* | *-* | *-* | n/a |
| SCA34 | *-* | *6p12.3-q16.2* | *chr6:46,200,000 - 100,600,000* | *chr12* | n/a |
| SCA35 | *TGM6* | *20p13* | *chr20:2,361,554-2,413,399* | *chr24:21,697,299-21,709,977* | Insufficient read depth to analyse |
| SCA36 | *NOP56* | *20p13* | *chr20:2,633,178-2,639,039* | *chr24:21,559,606-21,563,511* | No polymorphisms identified |
| SCAR1 | SETX | 9q34.13 | chr9:135,136,827-135,230,372 | chr9:55,244,347-55,323,850 | Heterozygous SNPs identified - gene excluded |
| SCAR2 | *-* | *9q34-qter* | *chr9:137,919,616-138,285,463* | *chr9:53,000,000-55,000,000* | n/a |
| SCAR3 | *-* | *6p23-p21* | *chr6:13,400,000 - 46,200,000* | *chr35,chr12* | n/a |
| SCAR4 | *-* | *1p36* | *chr1:3,584,862-15,028,985* | *chr5,chr2* | n/a |
| SCAR5 | *ZNF592* | *15q25.3* | *chr15:85,291,818-85,349,663* | *chr3:56,896,457-56,914,856* | No coding polymorphism identified |
| SCAR6 | *-* | *20q11-q13* | *chr20:19,831,375-43,649,175* | *chr23,chr24* | n/a |
| SCAR7 | *-* | *11p15* | *chr11:2754932-7292350* | *chr18,chr21* | n/a |
| SCAR8 | *SYNE1* | *6q25.1-q25.2* | *chr6:152,442,819-152,958,534* | *chr1:45,428,916-45,877,003* |  |
| SCAR9 | *ADCK3* | *1q42.13* | *chr1:227,127,938-227,175,246* | *chr7:41,056,946-41,076,326* | 1 non-synonymous SNP (p.S328P.) P is conserved among species |
| SCAR10 | *ANO10* | *3p22.1* | *chr3:43,407,818-43,663,560* | *chr23:5,692,957-5,911,487* | No polymorphisms identified |
| SCAR11 | *SYT14* | *1q32.2* | *chr1:210,111,538-210,337,633* | *chr7:11,556,473-11,746,853* | One synonymous SNP identified |
| SCAR12 | *-* | *16q21-q23* | *chr16:65,067,301-82,980,450* | *chr5:71,732,917-87,083,276* | n/a |
| Friedrich ataxia | *FXN* | 9q21.11 | chr9:71,650,479-71,715,094 | chr1:91,355,667-91,378,319 | One synonymous SNP identified |
