# Supplementary material for: Genome-wide mRNA sequencing of a single canine cerebellar cortical degeneration case leads to the identification of a disease associated SPTBN2 mutation
Source: BMC Genet. 2012 Jul 10;13:55. doi: 10.1186/1471-2156-13-55 (PMC3413603; doi:10.1186/1471-2156-13-55)
Supplement: Additional file 3 — Relative expression analysis data. Expression levels of SPTBN2 were measured relative to ACTB and TBP using qPCR. Fold change was calculated based on changes in threshold cycle (Ct) measurements within (ΔCt) and between (ΔΔCt) the case and control. [file 1471-2156-13-55-S3.doc]

**Additional file 3**

Relative expression analysis data. Expression levels of *SPTBN2* were measured relative to *ACTB* and *TBP* using qPCR. Fold change was calculated based on changes in threshold cycle (Ct) measurements within (∆Ct) and between (∆∆Ct) the case and control.

| **Sample Name** | **Assay Name** | **Ct Mean** | **Std Dev.** | **∆Ct** | **∆∆Ct** | **Fold ∆** | **Mean Fold ∆** |
| --- | --- | --- | --- | --- | --- | --- | --- |
| CONTROL | ACTB | 21.54 | 0.01 | 2.14 |  |  |  |
| CONTROL | TBP | 24.34 | 0.01 | -0.65 |  |  |  |
| CONTROL | SPTBN2 | 23.69 | 0.07 |  |  |  |  |
| BEAGLE | ACTB | 17.54 | 0.03 | 8.44 | 6.30 | 79 x |  |
| BEAGLE | TBP | 21.12 | 0.05 | 4.86 | 5.52 | 46 x | 68 x |
| BEAGLE | SPTBN2 | 25.98 | 0.05 |  |  |  |  |
